# Influence of larval growth and habitat shading on retreatment frequencies of biolarvicides against malaria vectors

**DOI:** 10.1038/s41598-024-51152-1

**Published:** 2024-01-10

**Authors:** Betwel J. Msugupakulya, Swedi K. Ngajuma, Athuman N. Ngayambwa, Baraka E. Kidwanga, Ibrahim R. Mpasuka, Prashanth Selvaraj, Anne L. Wilson, Fredros O. Okumu

**Affiliations:** 1 Environmental Health and Ecological Sciences Department, Ifakara Health Institute, P.O. Box 53, Ifakara, Tanzania; 2 Department of Vector Biology, Liverpool School of Tropical Medicine, Liverpool, UK; 3 Institute for Disease Modeling, Bill and Melinda Gates Foundation, Seattle, USA; 4 School of Life Science and Bioengineering, The Nelson Mandela African Institution of Sciences & Technology, Arusha, Tanzania; 5 School of Public Health, Faculty of Health Sciences, University of the Witwatersrand, Park Town, Republic of South Africa; 6 School of Biodiversity, One Health, and Veterinary Medicine, University of Glasgow, Glasgow, UK

**Keywords:** Malaria, Ecological epidemiology

## Abstract

Effective larviciding for malaria control requires detailed studies of larvicide efficacies, aquatic habitat characteristics, and life history traits of target vectors. Mosquitoes with brief larval phases present narrower timeframes for biolarvicidal effects than mosquitoes with extended periods. We evaluated two biolarvicides, VectoBac (*Bacillus thuringiensis israelensis* (*Bti*)) and VectoMax (*Bti* and *Bacillus sphaericus*) against *Anopheles funestus* and *Anopheles arabiensis* in shaded and unshaded habitats; and explored how larval development might influence retreatment intervals. These tests were done in semi-natural habitats using field-collected larvae, with untreated habitats as controls. Additionally, larval development was assessed in semi-natural and natural habitats in rural Tanzania, by sampling daily and recording larval developmental stages. Both biolarvicides reduced larval densities of both species by >98% within 72 h. Efficacy lasted one week in sun-exposed habitats but remained >50% for two weeks in shaded habitats. *An. funestus* spent up to two weeks before pupating (13.2(10.4–16.0) days in semi-natural; 10.0(6.6–13.5) in natural habitats), while *An. arabiensis* required slightly over one week (8.2 (5.8–10.6) days in semi-natural; 8.3 (5.0–11.6) in natural habitats). The findings suggest that weekly larviciding, which is essential for *An.* *arabiensis* might be more effective for *An. funestus* whose prolonged aquatic growth allows for repeated exposures. Additionally, the longer residual effect of biolarvicides in shaded habitats indicates they may require less frequent treatments compared to sun-exposed areas.

## Introduction

Vector control tools, particularly insecticide-treated nets (ITNs) and indoor residual spraying (IRS) have contributed significantly to the fight against malaria, together with other factors such as effective case management, and improved socioeconomic status^1,2^. However, recent data indicates that these gains are stalling in several countries^1^. ITNs and IRS, in particular, are undermined by multiple challenges including logistical difficulties, human behaviors that increase biting risk^3–6^, insecticide resistance^7,8^, and shifts in mosquito behaviors that enable vectors to evade indoor interventions^9–12^. It is thus necessary to adopt complementary interventions to sustain the gains against malaria.

Larval source management (LSM) is an attractive additional strategy for malaria control and refers to the targeted management of mosquito aquatic habitats, to reduce the densities of larvae, pupae, and emergent adults^13,14^. LSM is an umbrella term encompassing habitat modification (a permanent change to the environment), habitat manipulation (a recurrent activity), larviciding, and biological control^13,14^. The intervention has multiple benefits over the current indoor insecticide-based interventions, namely ITNs and IRS. By targeting mosquitoes in their aquatic stages, LSM has the potential to control vectors capable of evading indoor vector control interventions, including outdoor-biting and outdoor-resting, opportunistic biters, early-morning and early-evening biting mosquitoes, and insecticide-resistant mosquitoes^15^. The strategy has historically been effective in controlling, and even eliminating malaria vectors in several countries, including Brazil, Egypt (Upper Egypt), and Zambia (Copper Belt)^16–18^. However, despite its potential in reducing malaria transmission, LSM strategies have seen limited application in sub-Saharan Africa. The World Health Organization (WHO) recommends larviciding only as a supplementary control tool to ITNs and IRS, and only in places where habitats are fixed, few and findable, while habitat modification, habitat manipulation and biological control are not recommended by WHO^14^.

Chemicals such as dichlorodiphenyltrichloroethane, copper (II) acetate triarsenite (Paris green), and oil have historically been used to control mosquito larvae^16,17,19,20^. However, the majority of chemical larvicides are harmful to non-target organisms, including humans, and thus have been discontinued. Currently, only a few chemical larvicides are recommended for use in vector control, including insect growth regulators such as Pyriproxyfen, and organophosphates such as Temephos, and Spinosad^21^. However, these may compound insecticide resistance or impact non-target organisms^13,22^. Another option is biolarvicides derived from the microbial toxins, *Bacillus thuringiensis* var *israeliensis* (*Bti*) and *Bacillus sphaericus*, which are specific to mosquitoes and black flies^23^. These biolarvicides also have complex modes of action different from chemical insecticides, which may delay resistance development^23^. Nevertheless, mosquito larvae may react variably to bacterial larvicides^24^, as they do to many other insecticides, which may influence the outcome of larviciding programs.

Successful larviciding requires careful planning and management of different aspects of the programs^13,25^. Key considerations may include the choice of larvicide product itself, its efficacy and the residuality of the effect, and how this is impacted by different habitat types and environmental conditions. Although less considered in most larviciding programs, it is also important to understand the rate of egg-laying and larval growth dynamics of the vector species being targeted. Egg-laying and larval growth dynamics are particularly important for biolarvicides with short residual effect of less than one week and which target immature mosquitoes when in the feeding stages through ingestion of toxins. A retreatment interval of one week is often used for short-acting biolarvicides because the larval phase of *Anopheles* mosquitoes is assumed to be seven days^13,25,26^. However, laboratory studies indicate different larval periods for different *Anopheles* species, for example between 10–15 days for *An. funestus* and between 9–11 days for *An. gambiae*^27,28,39,40^. Given these differences, understanding the larval development period is therefore especially important for planning and optimizing the retreatment frequencies of biolarvicides. Moreover, the efficacy of biolarvicides can be influenced by various factors, such as exposure to sunlight, which can reduce the persistence of biolarvicides in aquatic habitats and potentially have an adverse effect on larviciding programs^29^. As *Anopheles* mosquito habitats can vary between shaded and sun-exposed^30,31^, the understanding of how this exposure may impact the efficacy of biolarvicides will help to choose appropriate application strategies for different vector species.

In preparation for a field trial in south-eastern Tanzania, this study, therefore, assessed the larvicidal effectiveness and residual activity of two biolarvicides, VectoBac GR and VectoMax FG (both manufactured by Valent Biosciences, Libertyville, IL 60048 USA) against two dominant malaria vector species, *An. funestus* and *An. arabiensis*, in shaded and un-shaded semi-natural aquatic habitats. We also evaluated the larval period of *An. funestus* and *An. arabiensis* under semi-natural and natural conditions.

## Results

### Effects of the biolarvicides on mosquito larvae in sun-exposed and shaded habitats

*Effects on late instar larvae in sun-exposed habitats at different biolarvicide doses*: Both VectoBac and VectoMax at different treatment rates of these two biolarvicides (5.6 kg/ha, 11.2 kg/ha, 22.4 kg/ha), resulted in 98.5–100% cumulative reduction of late instars of both *An. funestus* and *An. arabiensis* by the third day in all treated habitats. There was no significant difference in the cumulative mortality between habitats treated with different biolarvicides nor doses in the first three days after treatment for both species (p > 0.05). One week later, the effect of the biolarvicides diminished. The cumulative reduction in late instar *An. funestus* was 6.5% for VectoBac at 5.6 kg/ha on day 10, with cumulative larval mortality not significantly different from control (p > 0.05) (Table 1). *Anopheles funestus* habitats treated with VectoBac at 11.2 kg/ha and 22.4 kg/ha showed a cumulative reduction of late instars of 24.3% and 22.1%, respectively on day 10. While cumulative larval mortality of *Anopheles funestus* habitats treated with VectoBac at 11.2 kg/ha and 22.4 kg/ha was significantly higher than control on day 10 (p < 0.05), there was no significant difference between the two doses (p > 0.05). *An. funestus* habitats treated with VectoMax at 5.6 kg/ha, 11.2 kg/ha and 22.4 kg/ha showed 5.4%, 9.7% and 0% cumulative reduction on day 10, there was no significant difference between the two doses (p > 0.05). There was no residual efficacy of either larvicide at any dose against *An. arabiensis* by day 10, and habitats treated with VectoMax at 5.6 kg/ha and VectoBac at 11.2 kg/ha had less cumulative mortality (p < 0.05) while the cumulative mortalities in rest of treated habitats not significantly different from control habitats (p > 0.05).

**Table 1:** Efficacy of two biolarvicides (VectoBac GR and VectoMax FG) against *An. funestus* and *An. arabiensis* larvae in sun-exposed habitats.

Species	Biolarvicides (amount added per habitats)	Dose rate (kg/ha)	Week 1			Week 2		
			Number of larvae added per habitat in Day-1	Average No. live larvae remaining in Day-3	Percent reduction (%)	Number of larvae added in each habitat in Day-7	Average No. live larvae remaining in Day-10	Percent reduction (%)
Larval reduction of late instars (L3-L4) *An. funestus* and *An. arabiensis* in sun-exposed habitats at different treatment rate of the biolarvicides								
*An. funestus*	Control		45	40.2		35	30.7	
	VectoBac (73 mg)	5.6	45	0	100(100–100)	35	28.7	6.5(−1.9–14.9)
	VectoBac (146 mg)	11.2	45	0	100(100–100)	35	23.3	24.3(20.4–28.2)
	VectoBac (291 mg)	22.4	45	0	100(100–100)	35	23.9	22.1(14.7–29.5)
	VectoMax (73 mg)	5.6	45	0.5	98.7(97.8–99.6)	35	29.1	5.4(1.2–9.6)
	VectoMax (146 mg)	11.2	45	0.3	99.2(98.6–99.8)	35	27.7	9.7(2.8–16.6)
	VectoMax (291 mg)	22.4	45	0.6	98.5(97.6–99.4)	35	32.2	0
*An. arabiensis*	Control		80	68.3		70	63.5	
	VectoBac (73 mg)	5.6	80	0.8	98.8(98–99.6)	70	67.6	0
	VectoBac (146 mg)	11.2	80	0.5	99.2(98.6–99.8)	70	66.4	0
	VectoBac (291 mg)	22.4	80	0	100(100–100)	70	65.6	0
	VectoMax (73 mg)	5.6	80	0.1	99.9(99.7–100)	70	68.9	0
	VectoMax (146 mg)	11.2	80	0.5	99.3(98.8–99.8)	70	64.3	0
	VectoMax (291 mg)	22.4	80	0.5	99.2(98.6–99.8)	70	64.3	0
Larval reduction of early instars (L1-L2) *An. funestus* and *An. arabiensis* in sun-exposed habitats								
*An. funestus*	Control		60	53.8		ND	–	–
	VectoBac (130 mg)	10	60	0	100(100–100)	ND	–	–
	VectoMax (130 mg)	10	60	0	100(100–100)	ND	–	–
*An. arabiensis*	Control		25	20.5		ND	–	–
	VectoBac (130 mg)	10	25	0	100(100–100)	ND	–	–
	VectoMax (130 mg)	10	25	0	100(100–100)	ND	–	–

*ND* Not done.

*Effects on early instar larvae in sun-exposed habitats*: In a different experiment using 10 kg/ha of VectoBac and VectoMax, the cumulative reduction of early instars (L1-L2) was 100% for both *An. funestus* and *An. arabiensis* by the third day in sun-exposed habitats, with no significant difference between the two larvicides (p > 0.05) (Table 1). No observation was made on the tenth day after habitat treatment.

*Effects on late instars in shaded habitats*: In the shaded environment, habitats treated with VectoMax at an application rate of 11.2 kg/ha had a 100% cumulative reduction of late instars of both *An. funestus* and *An. arabiensis* within three days. On the tenth day, the larval reduction was still 63.3% for *An. funestus* and 57.9% for *An. arabiensis*. However, by the seventeenth day, the larval reduction was only 4.5% for *An. funestus* and 4.4% for *An. arabiensis* in the treated habitats (Table 2). The cumulative larval mortality on the third and tenth day after the treatment was significantly higher in the treated habitats of both *An. funestus* and *An. arabiensis* compared to control habitats (p < 0.05). On day 17, there was no significant difference in cumulative larval mortality between control and treated habitats (p > 0.05).

**Table 2:** Efficacy of VectoMax FG biolarvicide against *An. funestus* and *An. arabiensis* larvae in shaded habitats.

Time	Biolarvicides (amount added per habitats)	*An. funestus*			*An. arabiensis*		
		Number of larvae added per habitat	Average No. live larvae remaining 3 days later	Percent reduction (%)	Average No. larvae added (Day-1)	Average No. live larvae (Day-3)	Percent reduction (%)
Week 1	Control	45	41		50	48.7	
	VectoMax (146 mg)	45	0	100(100–100)	50	0	100(100–100)
Week 2	Control	85	80.9		100	97.2	
	VectoMax (146 mg)	85	29.7	63.3(49.5–77.1)	100	40.9	57.9(41–74.8)
Week 3	Control	50	47.3		50	47.1	
	VectoMax (146 mg)	50	45.1	4.5(0.6–8.4)	50	45	4.4(−1.2–10)

### The larval development period of *An. funestus* and *An. arabiensis* in the natural habitats

**Figure 1:**
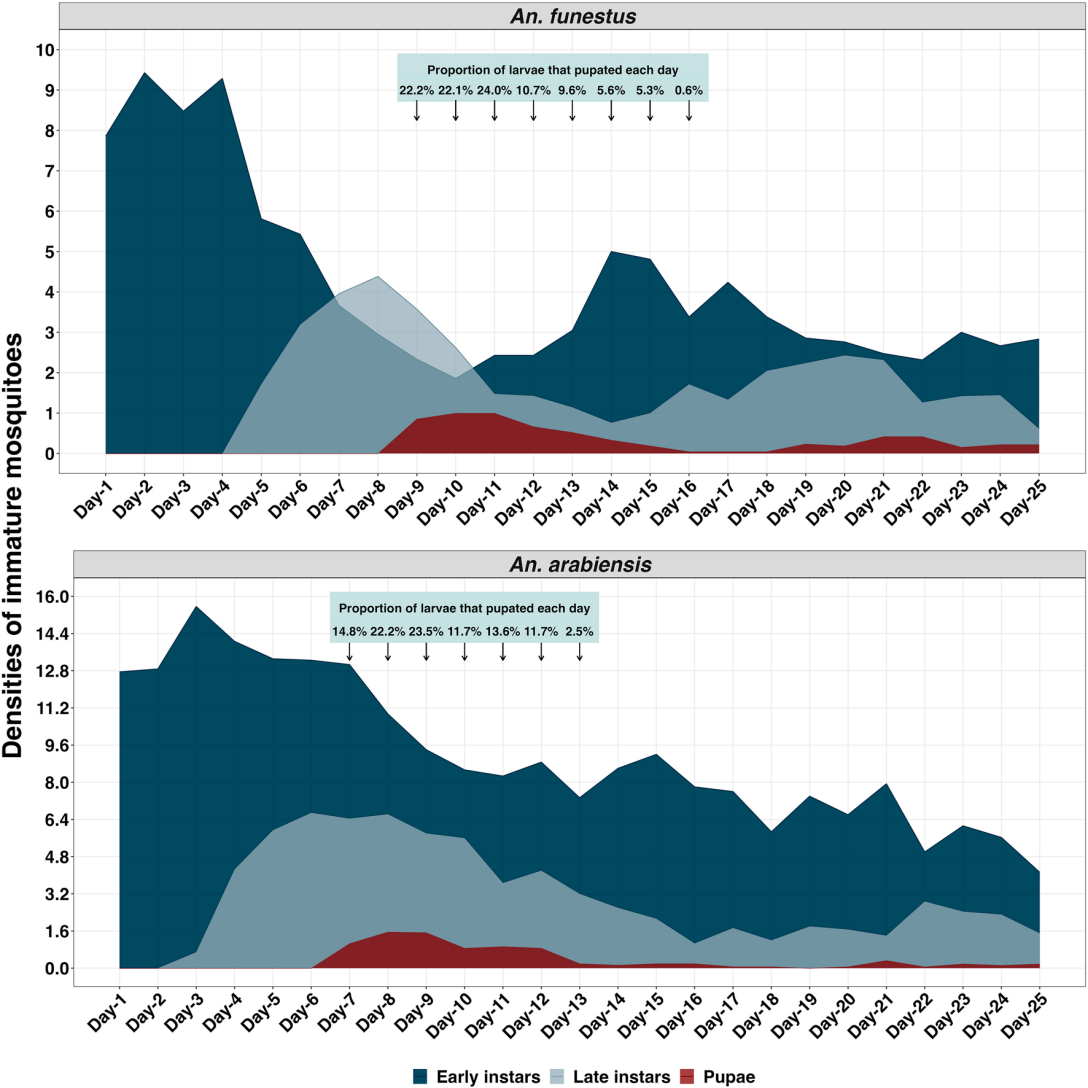
Dynamics of larvae and larval period of *An. funestus* and *An. arabiensis* in the natural habitats.

In the natural habitats that had only first-instar larvae at the start of the observation, the shortest period of larval development between the first and fourth instars was eight days for *An. funestus* and six days for *An. arabiensis* (assuming that all observed pupae had taken a least one day to transition from the fourth instar larval forms). The estimated mean larval development period in natural habitats was 10.0 (CI: 6.6–13.5) days for *An. funestus* and 8.32 (CI: 5.0–11.6) days for *An. arabiensis*. The range of the larval period was 8–15 days for *An. funestus* and 6–12 days for *An. arabiensis*. Larval stage duration varied among *Anopheles* species, with the 79% of *An. funestus* spending 8–11 days and 21% spending 12–15 days. The majority (85.8%) of *An. arabiensis* spent 6–12 days and 14.2% spent 13–14 days in the larval stage before pupating (Fig. 1).

Our assessment revealed a persistent presence of early instars of both *An. funestus* and *An. arabiensis* in their respective habitats. However, the population of all immature stages of *An. arabiensis* exhibited a continuous decline over the observation period. In contrast, the *An. funestus* population experienced a resurgence during the second week of observation, followed by a subsequent decline (Fig. 1).

### The larval development period of *An. funestus* and *An. arabiensis* in the semi-natural habitats

**Figure 2:**
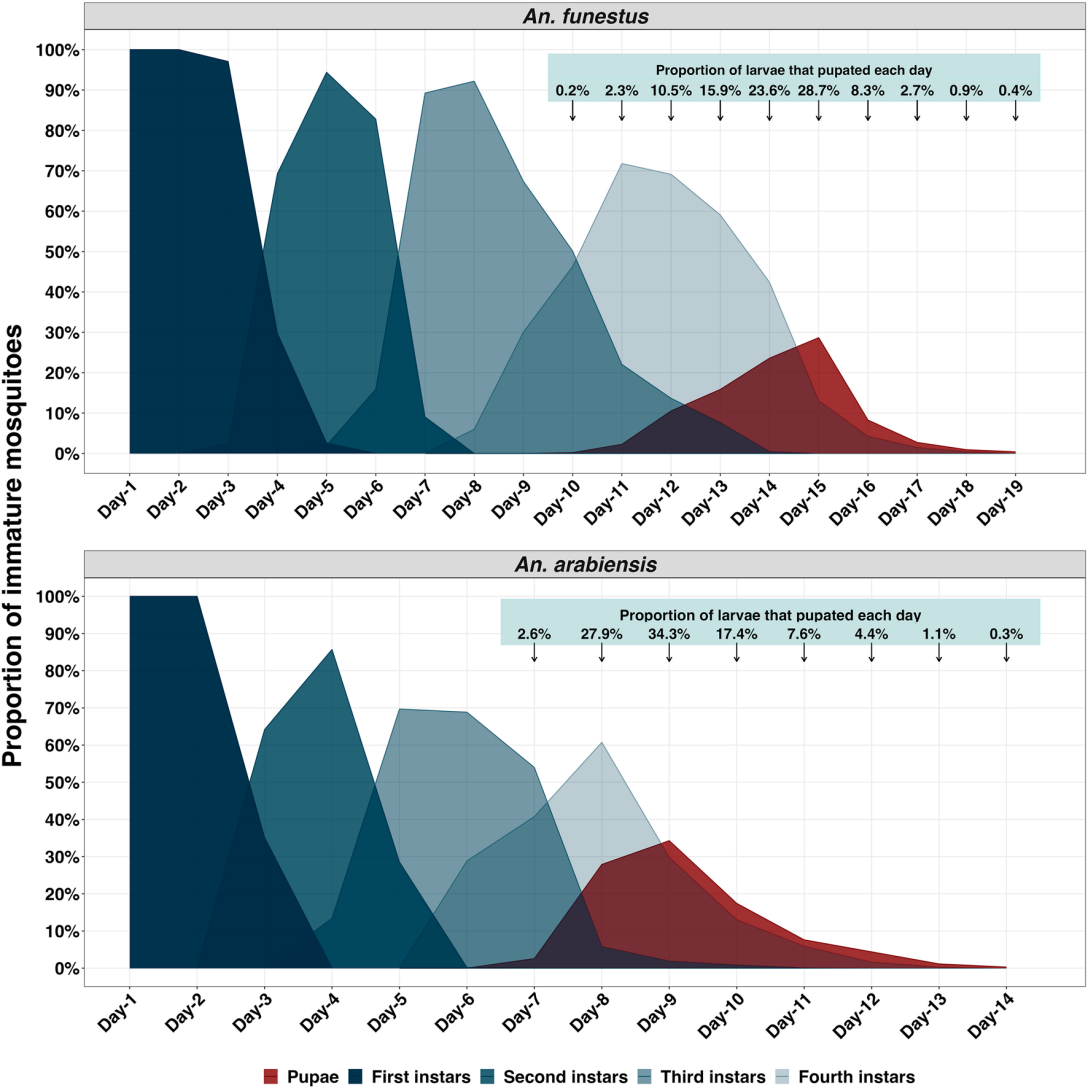
Larval period of *An. funestus* and *An. arabiensis* in the semi-natural habitats.

Assuming all observed pupae had taken at least one day to transition from the fourth instar larval forms, the shortest period of larval development between the first and fourth instars in the semi-natural habitats was nine days for *An. funestus* and six days for *An. arabiensis*. In the semi-natural habitats, the estimated mean larval development period was 13.2 (CI: 10.4–16.0) days for *An. funestus* and 8.2 (CI: 5.8–10.6) days for *An. arabiensis*. The range of the larval period was 9–18 days for *An. funestus* and 6–13 days for *An. arabiensis*. The majority (64.6%) of *An. funestus* spent 13–18 days as larvae, only 28.9% of *An. funestus* spent 9–12 days in larval stages, and the remaining (6.5%) died during the larval stage. The majority (89.8%) of *An. arabiensis* spent 6–10 days as larvae, only 5.8% of *An. arabiensis* spent 9–12 days in the larval stages, and the remainder (4.4%) died in the larval stage (Fig. 2).

## Discussion

This study investigated the efficacy and residual activity of two WHO-prequalified biolarvicides, VectoBac GR and VectoMax FG^21^, against the two major malaria vectors in Tanzania, *An. arabiensis* and *An. funestus* in habitats that were either shaded or unshaded. The larval development period of each species was also determined. The data are considered necessary to inform potential larviciding dosing and retreatment intervals for future studies.

Overall, this study found that VectoBac GR and VectoMax FG had very high efficacies (>98%) against both early and late instars of *An. funestus* and *An. arabiensis* within 72 h of habitat treatment, with no discernible difference between the two products. There was similarly high efficacy across the three different doses tested (5.6 kg/ha, 11.2 kg/ha, 22.4 kg/ha) within 72 h of treatment. However, both biolarvicides had short residual effects in sun-exposed habitats, lasting just one week. Increasing the treatment dose in sun-exposed habitats did not increase the residual efficacy of biolarvicides. However, residual efficacy in the second week was observed in shaded habitats treated with VectoMax, with approximately 60% reduction of L3-L4 of *An. arabiensis* and *An. funestus*. This suggests that sunlight might have likely reduced the residual efficacy of tested biolarvicides. These findings are consistent with previous research in Sub-Saharan Africa that reported low residual efficacy of biolarvicides in sun-exposed habitats^29,32–34^, which is a result of photoinactivation of the biolarvicide toxins^35^. This is a limitation of biolarvicides and may have implications for their use in Tanzania and elsewhere since not all habitats are shaded or vegetated^30,31^.

The estimated minimum and mean larval development period were similar for *An. arabiensis* in natural [minimum 6 days, mean 8.32 (CI: 5.0–11.6) days] and semi-natural habitats [minimum 6 days, mean 8.2 (CI: 5.8–10.6) days]. There were slight differences for *An. funestus*, which had a minimum of 8 days and a mean of 10 days (CI: 6.6–13.5) in natural habitats and a minimum of 9 days and a mean of 13.2 days (CI: 10.4–16.0) in semi-natural habitats. Factors such as temperature, food, and predators may affect larval growth^36–38^ and could have led to the differences in larval development time of *An. funestus* between the semi-natural and natural habitats. The observation that *An. funestus* has a longer larval development period than members of *An. gambiae* complex is similar to that reported in previous laboratory studies. These previous studies suggested a larval development period of 10–15 days for *An. funestus* and 9–11 days for *An. gambiae* ^27,28,39,40^.

The assessment of larval development in natural habitats was initiated with the first instars found in the habitats. Since we did not know their exact time after hatching, several first-instar larvae might likely have already spent more than one day in the natural habitats before we started our assessments, which could have led to an underestimation of the larval development period. Since early instar larvae were constantly observed in the natural habitats, despite the development of larvae, this indicated that new oviposition events were frequent in the study area. Thus, effectively targeting such habitats requires that the larviciding frequency does not exceed the larval development period of the target vectors.

Taken together, the findings of this study suggest that biweekly application of biolarvicides may be appropriate for settings where *An. funestus* dominates transmission including our study site due to the longer larval development time of the vector and preference for shaded habitats, such as those with vegetation or under tree canopies^30,31,41,42^. We may consider biweekly treatment a minimum interval, however, since it is likely that more frequent applications for *An. funestus* would increase impact. On the other hand, *An. gambiae* larval habitats may require weekly treatment with biolarvicides due to the shorter larval period and the higher likelihood of these habitats being sun-exposed^41,42^. These assertions could be explored using mathematical modeling, which could provide a quick way to understand the impact of different retreatment intervals before deployment.

Our study used biolarvicides that are relatively short-acting, however, longer-acting biolarvicides such as LL3 and FourStar are also available, which require less frequent retreatment and for which larval development time will play a lesser role^43^. A further consideration is the dynamics of habitat formation. Some authors recommend weekly application in areas with dynamic habitats and during the rainy seasons to ensure an effective dose even for new temporary habitats that form and cite this as being crucial for the larviciding team to get familiar with the target area^25^. While this is true, it may not be practical nor effective to implement larviciding during the rainy season as habitats are typically numerous and unstable, and larvicides are more likely to be diluted and washed away by rainwater^25^. The implementation of larviciding may be more feasible and effective when habitats are fixed, few, and findable, which in several areas may occur during dry season^13,14^, because during this season few new habitats form, and dilution or washing away of larvicides is not expected. Besides, the decline in populations of immature mosquitoes in natural habitats observed in this study, particularly for the *An. arabiensis* experiment (which was conducted later into the dry season) implies that the densities of immature mosquitoes decline over time during the dry season. Therefore, larval control efforts during this period may be more effective than in wet seasons and would add further stress to the already declining population.

Although successful, this study was not without limitations. First, there are several factors, including the physicochemical parameters of water, which we did not measure, and which may have affected the efficacy of biolarvicides^23^. Second, this study was conducted in semi-natural habitats made of plastic containers, which may not reflect the conditions of aquatic habitats in the field as most Afro-tropical *Anopheles* are not container breeders. Nevertheless, to minimize these potential effects, soil and vegetation were added to the habitats, habitats were sunk into the surrounding soil, and habitats were allowed to condition for several days so that the habitats could better mimic the field environment. Third, because dead larvae play a role in the regeneration of *B. sphaericus* spores in aquatic habitats it is likely that our procedure which involved the removal of dead larvae from the habitats when that was possible may have contributed to the reduced residual efficacy of VectoMax^45^. Lastly, the scope of this study is limited to frequencies and retreatment intervals of larviciding, however, an effective larviciding program would also require a proper plan to identify, treat and monitor aquatic habitats of the vectors^13,46^. Different retreatment strategies may have implications for efficacy, as suggested here, but also feasibility and cost depending on the available resources and expertise. In addition to being effective, larviciding programs must also be sustainable and acceptable. This requires strong collaboration with different stakeholders and local communities to incorporate their needs and recommendations^13^.

## Conclusions

This study investigated the efficacy and residual activity of two commercially available biolarvicides, VectoBac GR and VectoMax FG against two dominant malaria vector species, *An. funestus* and *An. arabiensis*, in shaded and un-shaded semi-natural aquatic habitats. We also evaluated the larval period of *An. funestus* and *An. arabiensis* under semi-natural and natural conditions. The findings revealed high biolarvicidal efficacy exceeding 98% within three days of treatment for both vectors. At day 3 there was no significant difference in efficacy observed between the two products or different doses in the manufacturer recommended range. However, the persistence of this effect was transient in sun-exposed habitats, lasting merely a week. In contrast, larviciding in shaded habitats with VectoMax demonstrated prolonged efficacy. The diminished biolarvicide longevity in the sun-exposed environment indicates that sun-exposed habitats such as those preferred by species like *An. arabiensis* may require more frequent treatments. Regarding the larval development, *An. funestus* required up to two weeks to reach the pupal stage, whereas *An. arabiensis* took just over a week. This difference in growth speeds suggests the possibility of differential treatment frequencies: i.e., a minimum of biweekly for *An. funestus* and a weekly minimum for the faster-developing *An. arabiensis*. However, it is likely that more frequent applications for *An. funestus* might maximize impact. Furthermore, the consistent presence of early instars in natural habitats points to regular oviposition events, emphasizing the importance of tailoring larviciding schedules to the larval development timeframes of the targeted vectors. Collectively, these insights underline the multi-faceted considerations crucial for optimizing larviciding interventions.

## Methods

### Study site and the aquatic habitats

This evaluation was conducted at the Ifakara Health Institute’s Mosquito City facility, which comprises large semi-field mesocosms, in Kining’ina village (8°06′28.8″ S 36°40′00.5″ E) in Ifakara Town Council, in south-eastern Tanzania. The rainy season occurs mainly in months between December and May, with the rest of the year being dry. The tests to evaluate the efficacy and residual efficacy of the biolarvicides were conducted in semi-natural aquatic habitats created inside and outside the semi-field systems to mimic the natural habitats of malaria vectors, following WHO procedures^49^. In the study area, *An. gambiae* s.l is known to consist almost entirely of *An. arabiensis*, while *An. funestus* group is composed mostly of *An. funestus* sensu stricto (s.s), with a very small proportion (<10%) of *An. leesoni*, and *An. rivulorum* ^31,50–53^. Therefore, in this manuscript, the mosquitoes are referred to simply as *An. arabiensis* and *An. funestus*.

**Figure 3:**
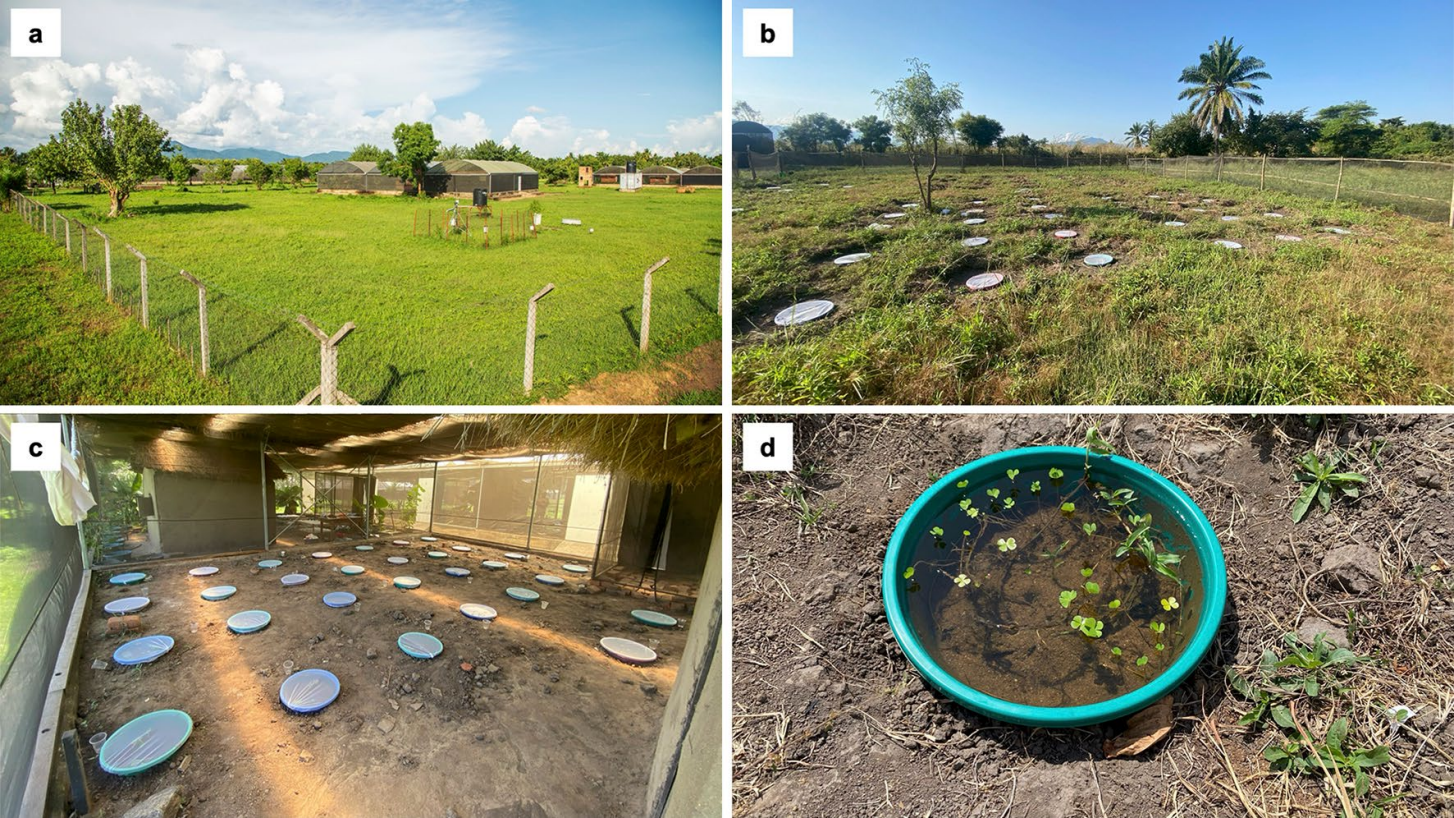
Semi-natural habitats created at Mosquito City (**a**). The habitats were created either outside the semi-field screen houses, i.e., open to direct sunlight (**b**), or inside the semi-field systems, i.e., shaded environment (**c**). (**d**) An aquatic habitat with the netting cover removed.

To simulate the aquatic habitats of *Anopheles* mosquitoes, pits measuring 50 cm (diameter) by 15 cm (depth) were dug in the open field (exposed to sunlight) or within the semi-field chambers (shaded). Plastic basins (41 cm diameter) were installed in the pits (Fig. 3). The plastic basins had first been filled with water and left for 3 days to wash away plastic odors, then the water was discarded before the basins were used for these experiments. Semi-natural conditions were created by adding a 1–2 cm layer of soil (collected from the natural aquatic habitats in local villages) to the basin and filling it with untreated groundwater (from a borehole at the Mosquito City) to a depth of ~13 cm. Vegetation from the same natural habitats in the villages was also added into the habitats for larvae anchoring. Untreated netting secured with elastic bands was used to cover each habitat to prevent emerging mosquitoes from flying out of these habitats, prevent other mosquitoes and insects from laying eggs in the semi-natural habitats, and prevent any outside debris from entering the containers. The habitats were left for a minimum of a week to allow any eggs of mosquitoes or predators that may have been present (in either the water, vegetation, or soil), to hatch; so that the larvae could be removed from the habitats before the initiation of the tests.

### Biolarvicide formulations

VectoBac (GR) and VectoMax (FG) granules (both from Valent Biosciences, Libertyville, IL 60048, USA) were evaluated. The VectoBac formulation contained 2.8% w/w of *Bacillus thuringiensis* subsp. *israeliensis* strain AM65-52. The VectoMax formulation contained a mixture of 4.5% w/w *Bacillus thuringiensis* subsp. *israeliensis* strain AM65-52 and 2.7% w/w *Bacillus sphaericus* 2362 strain ABTS-1743. VectoBac GR consisted of higher density granules (~689 kg/m^3^) with size approximately between 0.8–2.0 mm, while VectoMax consisted of moderate density granules (480 kg/m^3^) with size approximately between 1.4–2.0 mm. Manufacturer recommended application rates are 2.8–22.4 kg/ha for VectoBac and 5.6–22.4 kg/ha for VectoMax depending on factors including the density of mosquitoes, pollution or algae levels in the habitat.

### Mosquito larvae used for the tests

**Figure 4:**
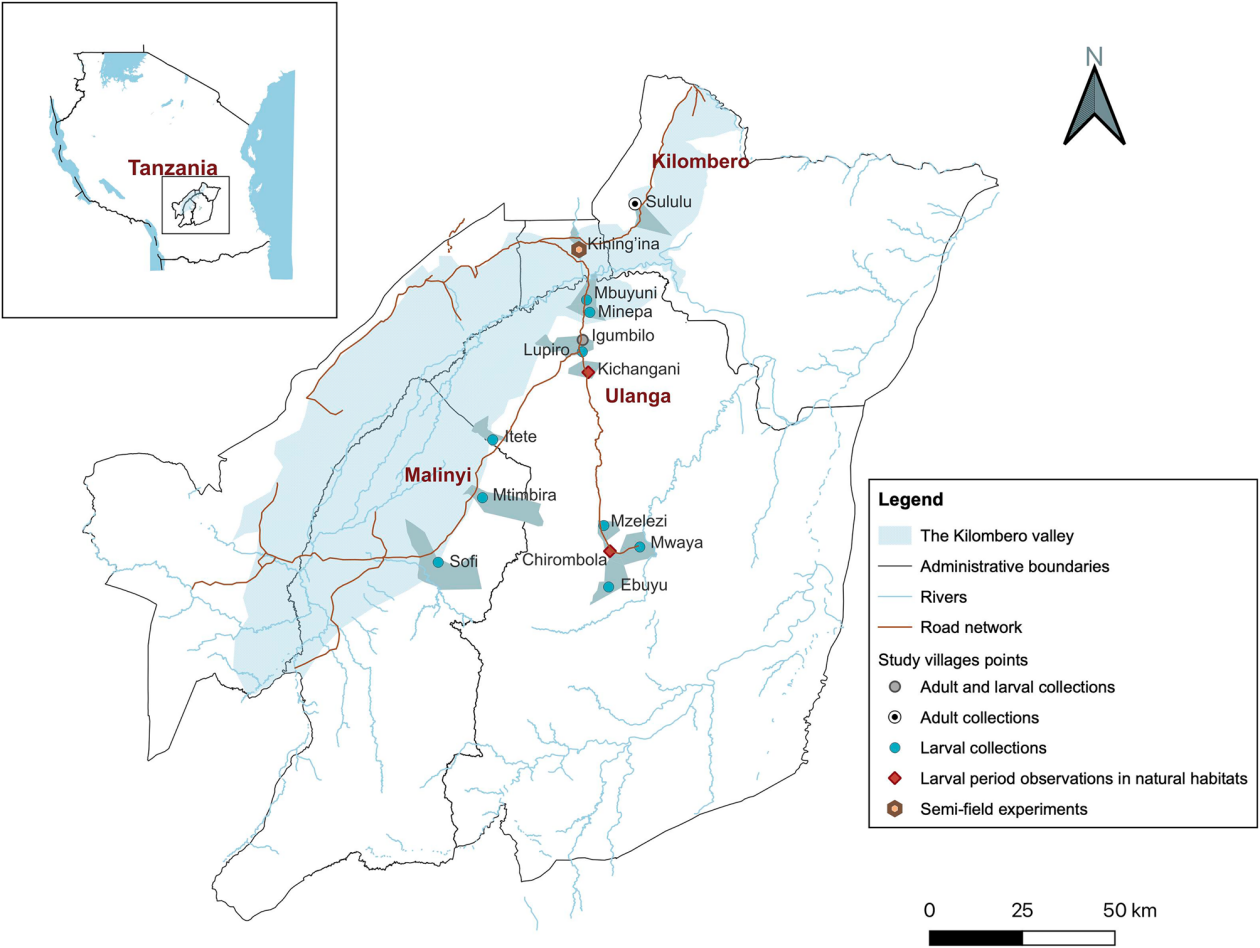
Study sites for the efficacy of the biolarvicides, collection of larvae and adult mosquitoes, and observations of larval periods in natural habitats.

The larvae used in this assessment were collected from rural Tanzanian villages. *An. funestus* larvae were collected from known aquatic habitats in Itete (8°58′60″ S 36°07′ E), Sofi (8°57′S 36°17′ E), Mtimbira (8°47′S 36°21′ E), Mzelezi (8°52′50″ S 36°43′50″ E), Ebuyu (8°58′20″ S 36°45′40″ E), and Mwaya (8°54′43″ S 36°49′53″ E) villages, while larvae of *An. arabiensis* were collected from known habitats in Mbuyuni (8°14′38″ S 36°41′15 E), Minepa (8°15′58″ S 36°41′05″ E), Igumbiro (8°21′ S 36°40′22″ E), Lupiro (8°23′ S 36°40′33″ E), and Mwaya villages (Fig. 4). *An. funestus* were collected using 10-liter buckets from large habitats such as ponds, wells, and streams, while *An. arabiensis* were collected using standard dippers (350 milliliters) from puddles and rice fields. Collected larvae were transferred to collection buckets and transported the same day to the insectary in Mosquito City, where they were sorted into their respective instars (L1-L4).

### Testing the efficacy and residual efficacy of biolarvicides

In this assessment, a total of 15 habitats were created for the treatment arm and another 15 for the control arm. The sample size was estimated using R statistical software^54^, based on previous observations^24^ and was sufficient to detect mortality of at least 69% in the treatment arm, with 80% power within a 95% confidence interval. The efficacy testing was conducted from July 2022 to February 2023. Each efficacy test was conducted on one occasion with 15 replicates (habitats) per arm.

Cohorts of mosquito larvae (between 20 and 150, depending on total number of larvae collected in the field) were added to each of the habitats and supplemented with larval food (Tetramin fish food). Efficacies of the biolarvicides against *An. funestus* and *An. arabiensis* were evaluated separately in different habitats. Larvae in the semi-natural habitats were allowed to acclimatize for at least three hours, after which, in the treatment arms, the biolarvicides were applied by hand. Three different experiments were performed, with treatment doses within the manufacturer's recommendations. No larvicides were added into habitats in the control arm. Tests were conducted in the sun-exposed and shaded environment to assess differences in the residual efficacy of biolarvicides under these two conditions (Table 3). However, in the shaded habitats, the residual effect was tested only for VectoMax because it contains *B. sphaericus*, which can persist in the environment.

**Table 3:** Treatment doses used in three different tests of efficacies of VectoBac and VectoMax using artificially created, semi-natural habitats.

Treatment arms	Amount of biolarvicides added in the habitats (0.13 m^2^)	Manufacturer’s equivalence
*Tests in the sun-exposed environment*		
i) Bioassays of VectoBac and VectoMax against late instars (L3 and L4) of *An. funestus* and *An. arabiensis* both at different dose rates in semi-natural habitats exposed to sunlight		
Control	0 mg	
VectoBac GR (73 mg) arm	72.8 mg	5.6 kg/ha
VectoBac GR (146 mg) arm	145.6 mg	11.2 kg/ha
VectoBac GR (291 mg) arm	291.2 mg	22.4 kg/ha
VectoMax FG (73 mg) arm	72.8 mg	5.6 kg/ha
VectoMax FG (146 mg) arm	145.6 mg	11.2 kg/ha
VectoMax FG (291 mg) arm	291.2 mg	22.4 kg/ha
ii) Bioassays of VectoBac and VectoMax against early instars (L1-L2) of *An. funestus* and *An. arabiensis* in semi-natural habitats exposed to sunlight		
Control	0 mg	
VectoBac GR (130 mg) arm	130 mg	10 kg/ha
VectoMax FG (130 mg) arm	130 mg	10 kg/ha
*Tests in the shaded environment*		
Bioassays of VectoMax against late instars (L3 and L4) of *An. funestus* and *An. arabiensis* in semi-natural habitats in the shaded area		
Control	0 mg	
VectoMax FG (146 mg) arm	145.6 mg	11.2 kg/ha

All habitats were observed for 24 h, 48 h, and 72 h (first, second, and third day) post-treatment, and the number of larvae and pupae in the habitats was counted by carefully transferring all live larvae into a disposable cup using a Pasteur pipette. The dipping was done exhaustively, ensuring that all live larvae were caught. The contents of the disposable cup were gently poured back into the appropriate habitat after counting. Clean pipettes and cups were used each time for different habitats to avoid contamination. It was difficult to find all the dead larvae, as some deteriorated quickly or became covered with soil or vegetation in the habitat, and as such we counted live larvae and presumed that any decline in number was the result of larval mortality. Seven days from the first exposure, another cohort of larvae (between 20 and 150, depending on the total number of larvae collected in the field) was introduced in each aquatic habitat without any addition of biolarvicides, and observations of larvae were made at 24, 48, and 72 h. This procedure was repeated seven days from the previous larval exposure until the recoverable live larvae in the treatment habitats were comparable to the control.

### Assessing the larval development period in natural habitats

Eleven natural habitats of *An. funestus* and 12 habitats of *An. arabiensis* with high densities of first instar larvae were identified in Kichangani (8°25′ 10″ S 36°40′51″ E) and Chirombola (8°55′35″ S 36°45′14″ E) villages; and used to assess the time spent by larvae from the first to fourth instars.

These observations were made between May and August 2023 (dry season). To ensure that the observations in natural habitats started with first instars, exhaustive dipping was done in the selected habitats using 10-L buckets to identify all instar stages present in the habitats. Any second or higher-instar larvae detected were removed and discarded, so that only first-instars remained in the habitats. This process was repeated until the dips taken produced only the first instars. No efforts were made to remove any eggs that might have been in the habitats.

Habitats were left uncovered to allow mosquitoes to continue laying eggs in them. To assess their development, larvae in the habitat were monitored daily. Depending on the size of the habitats, one to five dips were taken from each habitat using a 10-liter bucket. From each habitat, dips were taken from different locations to minimize sampling similar larvae on different dipping attempts. The number and instar stages of mosquitoes from each dip were recorded for estimation of larval densities (early vs. late instars), after which the larvae were carefully placed back in the habitat. This experiment allowed the assessment of the minimum larval period of *An. funestus* and *An. arabiensis* in the natural habitats, as well as the determination of whether oviposition for each species was occurring frequently or in batches.

### Assessing the larval development period in semi-natural habitats

Assessment of the larval development period in semi-natural habitats was conducted using eggs laid by field-collected mosquitoes. These experiments were conducted between April and May 2023. Blood-fed and unfed indoor-resting and host-seeking *An. funestus* and *An. arabiensis* were collected from Sululu (7°59′ S 36°49′ E) and Igumbiro (8°21′ S 36°40′22″ E) villages (Fig. 4), and live mosquitoes were transferred on the same or second day to the insectary at Ifakara Health Institute. Mosquitoes were placed in 15×15 cm cages, and unfed mosquitoes were fed on chicken blood. All mosquitoes were maintained with a 10% sucrose solution. On the fourth day, mosquitoes were individually transferred to paper cups with wet filter paper for egg laying. Eggs were allowed to hatch before placing them in the habitats. For this experiment, 20 semi-natural habitats were created in the sun-exposed area using submerged plastic basins with habitat soil and plants added as described earlier. 80–110 first instar larvae were added into each habitat. *An. funestus* and *An. arabiensis* were placed in separate habitats (10 habitats for each species).

Larval growth was monitored daily by observing and recording the number of larvae per instar stage in each habitat. The number of larvae and pupae in each habitat was counted by carefully transferring all live larvae and pupae into a disposable cup using a Pasteur pipette. The contents of the disposable cup were gently poured back into the habitat after counting. The observations continued until all the mosquitoes pupated or died. Since the initial number of larvae was known, and no new larvae were introduced in the habitats, this experiment allowed the estimation of the larval development period, as well as the proportion of individual larvae with different larval development periods.

### Identification of mosquito species

Sub-samples of field-collected *Anopheles* mosquitoes were taken from each experiment and reared to adults for subsequent identification. In both experiments emerging adult mosquitoes were identified morphologically to species^55^.

### Data analysis

Data was collected using ODK^56^, and, upon entry, the research team cross-checked it for any errors. Larval mortality was defined as the number of dead larvae/number of larvae added, with cumulative mortality calculated as the number of dead larvae on day 3, 10, or 17/number of larvae added. The efficacy of the biolarvicides was estimated as the percent reduction of live larvae in the intervention habitats relative to control habitats. The cumulative percent larval reduction (Lr) was estimated as, %Lr = *((Cp – Tp)/ Cp*) × 100, where *Cp* and *Tp* were the proportion of live larvae remaining in the control and treatment habitats on the third day after being added to the habitats, respectively. The proportion of live larvae was estimated by dividing the total number of larvae and pupa that survived by the total number of larvae added to the respective habitats. The mean cumulative percent reduction was then estimated with its 95% confidence intervals. Comparison of cumulative mortality of larvae between different of habitats treated or untreated with biolarvicides was made using the Kruskal-Wallis test followed by a pairwise Wilcoxon rank sum test with Bonferroni correction to determine which groups differed significantly from each other. Results with p-value < 0.05 were considered statistically significant.

Larval development time was estimated as the number of days lapsed when pupae were detected minus one (assuming that pupae observed were in their fourth instar on the previous day). The minimum larval period was estimated as the time taken till the first larva was pupated, while the maximum larval period was the time taken till the last pupa was observed. The proportion of total pupae observed in a specific day in habitats was estimated by comparing pupae density in that day to the total pupae density in a specific period of pupation (the specific period of pupation refers to the peak of pupal appearance, with secondary peaks observed in natural habitats presumed to be from another wave of egg laying). The mean larval periods were estimated using the following formula: *∑*(n*(*d*−1))/*N*, where ‘n’ is the density of larvae pupating on a specific day, ‘*d*−1’ is the larval duration, and ‘N’ is the total density of larvae that pupated in a wave and presented with a 95% confidence interval.

Data analysis was conducted in R software^54^, and plots were generated using the *ggplot2* package^57^.

### Ethics approval and consent to participate

Ethics approvals for this study were obtained from the Institutional Review Board of Ifakara Health Institute (Ref no: IHI/IRB/No: 32-2021) and the Medical Research Coordinating Committee (MRCC) at the National Institute for Medical Research (Ref no: NIMR/HQ/R.8a/Vol. IX/3761).

### Consent for publication

Permission to publish was granted by The National Institute for Medical Research (NIMR), Tanzania, Ref. No: BD.242/437/01/68.

## Data Availability

The datasets used and/or analyzed during the current study are available from the corresponding author upon reasonable request.

## Abbreviations

Bti: *Bacillus thuringiensis* Subsp. *israeliensis*
ID: Identifier
IRS: Indoor residual spraying
ITNs: Insecticide-treated nets
LSM: Larval source management
MS Excel: Microsoft Excel
WHO: World Health Organization

## References

[1] WHO. *World Malaria Report 2022* (2022).

[2] Bhatt S (2015). The effect of malaria control on *Plasmodium falciparum* in Africa between 2000 and 2015. Nature.

[3] Monroe A, Moore S, Koenker H, Lynch M, Ricotta E (2019). Measuring and characterizing night time human behaviour as it relates to residual malaria transmission in sub-Saharan Africa: a review of the published literature. Malar. J..

[4] Dunn CE, le Mare A, Makungu C (2011). Malaria risk behaviours, socio-cultural practices and rural livelihoods in southern Tanzania: Implications for bednet usage. Soc. Sci. Med..

[5] Finda MF (2019). Linking human behaviours and malaria vector biting risk in south-eastern Tanzania. PLoS ONE.

[6] Swai JK (2016). Studies on mosquito biting risk among migratory rice farmers in rural south-eastern Tanzania and development of a portable mosquito-proof hut. Malar. J..

[7] Ranson H, Lissenden N (2016). Insecticide resistance in African *Anopheles* mosquitoes: a worsening situation that needs urgent action to maintain malaria control. Trends Parasitol..

[8] Hemingway J (2016). Averting a malaria disaster: Will insecticide resistance derail malaria control?. Lancet.

[9] Govella, N. J., Chaki, P. P. & Killeen, G. F. Entomological surveillance of behavioural resilience and resistance in residual malaria vector populations. *Malar. J.* **12**, 1–9 Preprint at 10.1186/1475-2875-12-124 (2013). 10.1186/1475-2875-12-124 PMC3637503 23577656

[10] Russell, T. L., Beebe, N. W., Cooper, R. D., Lobo, N. F. & Burkot, T. R. Successful malaria elimination strategies require interventions that target changing vector behaviours. *Malar. J.* **12**, 1–5 Preprint at 10.1186/1475-2875-12-56 (2013). 10.1186/1475-2875-12-56 PMC3570334 23388506

[11] Moiroux N (2012). Changes in *Anopheles funestus* biting behavior following universal coverage of long-lasting insecticidal nets in Benin. J. Infect. Dis..

[12] Msugupakulya BJ (2020). Preferred resting surfaces of dominant malaria vectors inside different house types in rural south-eastern Tanzania. Malar. J..

[13] WHO. *Larval source management: a supplementary measure for malaria vector control* (2013).

[14] WHO. *Guidelines for Malaria Vector Control*. (2019). 30844152

[15] Killeen GF, Fillinger U, Knols BG (2002). Advantages of larval control for African malaria vectors: low mobility and behavioural responsiveness of immature mosquito stages allow high effective coverage. Malar. J..

[16] Soper, F. L. & Wilson, D. B. *Anopheles gambiae in Brazil, 1930 to 1940*. (Rockefeller Foundation, 1943).

[17] Shousha AT (1948). Species-eradication: The Eradication of *Anopheles gambiae* from Upper Egypt, 1942–1945. Bull. World Health Organ..

[18] Schumaker, L. The Mosquito Taken at the Beerhall: Malaria Research and Control on Zambia’s Copperbelt*. in *Evidence, Ethos and Experiment: The Anthropology and History of Medical Research in Africa* (eds. Geissler, P. W. & Molyneux, C.) (2011).

[19] Wilson, A. L. *et al.* The importance of vector control for the control and elimination of vector-borne diseases. *PLoS Negl. Trop. Dis.* **14**, e0007831 (2020). 10.1371/journal.pntd.0007831 PMC6964823 31945061

[20] Watson, M. *African Highway. The Battle for Health in Central Africa. African Highway. The Battle for Health in Central Africa.* (John Murray, 1953).

[21] WHO. Prequalified Vector Control Products | WHO - Prequalification of Medical Products (IVDs, Medicines, Vaccines and Immunization Devices, Vector Control). https://extranet.who.int/pqweb/vector-control-products/prequalified-product-list?field_product_type_tid=89&field_pqt_vc_ref_number_value=&title=&field_applicant_tid=&field_active_ingredient_synergis_tid= (2023).

[22] Opiyo, M. A. *et al.* Sub-lethal aquatic doses of pyriproxyfen may increase pyrethroid resistance in malaria mosquitoes. *PLoS ONE* **16**, e0248538 (2021). 10.1371/journal.pone.0248538 PMC7971891 33735241

[23] Lacey LA (2007). *Bacillus thuringiensis* serovariety *israelensis* and *Bacillus sphaericus* for mosquito control. J. Am. Mosq. Control Assoc..

[24] Derua, Y. A., Kweka, E. J., Kisinza, W. N., Githeko, A. K. & Mosha, F. W. Bacterial larvicides used for malaria vector control in sub-Saharan Africa: Review of their effectiveness and operational feasibility. *Parasites and Vectors* vol. 12 Preprint at 10.1186/s13071-019-3683-5 (2019). 10.1186/s13071-019-3683-5 PMC6716942 31470885

[25] Fillinger U, Lindsay SW (2011). Larval source management for malaria control in Africa: Myths and reality. Malar. J..

[26] Fillinger U (2008). A tool box for operational mosquito larval control: preliminary results and early lessons from the Urban Malaria Control Programme in Dar es Salaam. Tanzania..

[27] Munga, S., Vulule, J. & Kweka, E. J. Response of *Anopheles gambiae* s.l (Diptera: Culicidae) to larval habitat age in western Kenya highlands. *Parasit Vectors* **6** (2013). 10.1186/1756-3305-6-13 PMC3564891 23324330

[28] Ngowo, H. S., Hape, E. E., Matthiopoulos, J., Ferguson, H. M. & Okumu, F. O. Fitness characteristics of the malaria vector *Anopheles funestus* during an attempted laboratory colonization. *Malar. J.* **20**, (2021). 10.1186/s12936-021-03677-3 PMC7955623 33712003

[29] Zogo B (2019). Impact of sunlight exposure on the residual efficacy of biolarvicides *Bacillus thuringiensis israelensis* and *Bacillus sphaericus* against the main malaria vector, *Anopheles gambiae*. Malar. J..

[30] Debrah I (2021). Larval ecology and bionomics of *Anopheles funestus* in highland and lowland sites in western Kenya. PLoS ONE.

[31] Nambunga, I. H. *et al.* Aquatic habitats of the malaria vector, *Anopheles funestus* in rural south-eastern Tanzania. 10.21203/RS.3.RS-20420/V1 (2020). 10.1186/s12936-020-03295-5 PMC7310514 32576200

[32] Djènontin A (2014). Field efficacy of vectobac GR as a mosquito larvicide for the control of anopheline and culicine mosquitoes in natural habitats in Benin, West Africa. PLoS ONE.

[33] Majambere S, Lindsay SW, Green C, Kandeh B, Fillinger U (2007). Microbial larvicides for malaria control in The Gambia. Malar. J..

[34] Derua YA (2022). Laboratory and semi-field evaluation of the efficacy of *Bacillus thuringiensis* var. *israelensis* (Bactivec®) and Bacillus sphaericus (Griselesf®) for control of mosquito vectors in northeastern Tanzania. Curr. Res. Parasitol. Vector Borne Dis..

[35] Pusztai M (1991). The mechanism of sunlight-mediated inactivation of *Bacillus thuringiensis* crystals. Biochem. J..

[36] Tuno N (2018). An algal diet accelerates larval growth of *Anopheles gambiae* (Diptera: Culicidae) and *Anopheles arabiensis* (Diptera: Culicidae). J. Med. Entomol..

[37] Rejmánková, E., Grieco, J., Achee, N. & R. Roberts, D. Ecology of Larval Habitats. in *Anopheles mosquitoes* (ed. Manguin, S.) Ch. 13 (IntechOpen, 2013). 10.5772/55229.

[38] Roux O, Robert V (2019). Larval predation in malaria vectors and its potential implication in malaria transmission: An overlooked ecosystem service?. Parasites Vectors.

[39] Mouline K (2012). Physiology and development of the M and S molecular forms of *Anopheles gambiae* in Burkina Faso (West Africa). Med. Vet. Entomol..

[40] Schneider P, Takken W, Mccall PJ (2000). Interspecific competition between sibling species larvae of *Anopheles arabiensis* and *An. gambiae*. Med. Vet. Entomol. J..

[41] Gillies MT, Coetzee M (1987). A supplement to the Anophelinae of Africa south of the Sahara (Afrotropical Region).

[42] Gimnig JE, Ombok M, Kamau L, Hawley WA (2001). Characteristics of Larval Anopheline (Diptera: Culicidae) Habitats in Western Kenya. J. Med. Entomol..

[43] Kahindi SC (2018). Efficacy and persistence of long-lasting microbial larvicides against malaria vectors in western Kenya highlands. Parasit. Vectors.

[44] Afrane YA (2016). Evaluation of long-lasting microbial larvicide for malaria vector control in Kenya. Malar. J..

[45] Becker N, Zgomba M, Petric D, Beck M, Ludwig M (1995). Role of larval cadavers in recycling processes of *Bacillus sphaericus*. J. Am. Mosq. Contol Assoc..

[46] Tusting L (2016). Mosquito larval source management for controlling malaria. Cochrane Database Syst. Rev..

[47] Mukabana WR (2022). Drones for area-wide larval source management of malaria mosquitoes. Drones.

[48] Maheu-Giroux M, Castro MC (2013). Impact of community-based larviciding on the prevalence of malaria infection in Dar es Salaam, Tanzania. PLoS ONE.

[49] WHO. *Guidelines for laboratory and field testing of mosquito larvicides*. (2005). doi:Ref: WHO/CDS/WHOPES/GCDPP/2005.11.

[50] Kaindoa EW (2017). Interventions that effectively target *Anopheles funestus* mosquitoes could significantly improve control of persistent malaria transmission in south-eastern Tanzania. PLoS ONE.

[51] Swai JK (2019). Protecting migratory farmers in rural Tanzania using eave ribbons treated with the spatial mosquito repellent, transfluthrin. Malar. J..

[52] Finda MF (2018). Dramatic decreases of malaria transmission intensities in Ifakara, south-eastern Tanzania since early 2000s. Malar. J..

[53] Mapua SA (2022). Persistently high proportions of *Plasmodium*-infected *Anopheles funestus* mosquitoes in two villages in the Kilombero valley, South-Eastern Tanzania. Parasite Epidemiol..

[54] R Core Team. R: A Language and Environment for Statistical Computing. Preprint at (2019).

[55] Coetzee M (2020). Key to the females of Afrotropical *Anopheles* mosquitoes (Diptera: Culicidae). Malar. J..

[56] Hartung, C. *et al.* Open Data Kit: Tools to build information services for developing regions. In *Proceedings of the 4th ACM/IEEE International Conference on Information and Communication Technologies and Development* (Association for Computing Machinery, 2010). 10.1145/2369220.2369236.

[57] Wickham H (2011). ggplot2. WIREs Comput. Stat..

